# Demographics and help-seeking among significant others contacting the Swedish gambling helpline

**DOI:** 10.1177/14550725251320749

**Published:** 2025-03-25

**Authors:** David Forsström, Olof Molander, Anders Nilsson, Håkan Wall

**Affiliations:** Centre for Psychiatry Research, Department of Clinical Neuroscience, 27106 Karolinska Institutet, & Stockholm Health Care Services, Stockholm, Sweden; Centre for Psychiatry Research, Department of Clinical Neuroscience, 27106 Karolinska Institutet, & Stockholm Health Care Services, Stockholm, Sweden; Centre for Psychiatry Research, Department of Clinical Neuroscience, 27106 Karolinska Institutet, & Stockholm Health Care Services, Stockholm, Sweden; Centre for Psychiatry Research, Department of Clinical Neuroscience, 27106 Karolinska Institutet, & Stockholm Health Care Services, Stockholm, Sweden

**Keywords:** gambling, concerned significant others, help line, problems, help seeking

## Abstract

**Aims:** Concerned significant others (CSOs) to individuals that have gambling problems experience several negative consequences. Even though CSOs experience negative somatic and mental health consequences, there is an overall lack of research and available preventive or treatment strategies to help this vulnerable group. In Sweden, there is a helpline available to help individuals that gamble, but also helps concerned significant others. The aim of the present study was to investigate and describe facets of help seeking among CSOs who have called the Swedish national gambling helpline. **Methods:** The data was collected by personnel at the Swedish helpline. Descriptive statistics and chi-squared were used to analyze data. The sample consisted of a total of 4111 callers during a three-year period. **Results:** The results showed that CSOs constitute a diverse population with various needs for support. These needs can be linked to one's relationship with the individual who gambles (e.g. partners have different needs than parents). Moreover, CSOs have learned about the gambling problems in different ways and have known about the gambling problems for varying periods of time, which in turn affects what type of help they look for. **Conclusions:** The main implication of the study is that the CSO-group is diverse and has different needs when calling help services and different needs on an overall level. Prevention and treatment strategies need to adhere to this fact when creating different interventions to help this group.

## Introduction

Some individuals that engage in gambling develop problems that not only cause harm to them but also for concerned significant others (CSOs). These problems can also affect different types of significant others such as parents, partners, children, other relatives and friends (Irie & Kengo, 2022). It is not only severe gambling problems that cause harm. Low risk gambling can harm the individuals that gamble (Raisamo et al., 2014), as well as CSOs (Productivity Commission, 2010).

A global prevalence estimation of risky gambling is 1.4 % (Tran et al., 2024). Among young people, between 0.2% and 13% of individuals have some type of gambling problem (Calado et al., 2017). In Sweden, the prevalence of moderate risk gambling is 3–7 points on the problem gambling severity index (PGSI) (Ferris & Wynne, 2001), and problem gambling (8+ on the PGSI) is around 1.3%. People with increased risk make up about 0.6% of Sweden's population, whereas individuals who have a problematic gambling behavior make up about 0.7% of Sweden's population (Hofmarcher et al., 2020). One important aspect is that approximately 166,000 of the Swedish population that are concerned significant others are living with the 1.3% who have an increased risk and/or who have problems with gambling. Furthermore, approximately 410,000 of the Swedish population is living with an individual who has a gambling pattern associated with low risk (1–2 points on the PSGSI) (Hofmarcher et al., 2020). A review found that the prevalence of concerned significant others ranged from 2% to 19% depending on the methodology employed (Dowling et al., 2022). Other studies have found that, on average, six other individuals are negatively affected by one individual's problem gambling (Goodwin et al., 2017).

Different types of harm are present for gamblers, for concerned significant others and for society as a whole. An individual with an at-risk or problem gambling can suffer from a number of severe negative consequences. Langham et al. (2016) has proposed a taxonomy regarding different types of harm or negative consequences of gambling and identified seven different types. The harms/negative consequences that are suggested are: financial consequences, relationship problems, emotional and/or psychological harms, physical problems such as stress and heart disease, impact on work, studies or other activities involving financial gain, cultural consequences and criminal acts (Langham et al., 2016). Financial problems are the most common negative consequence. Having financial problems as a result of gambling can result in stress for the individual who gambles, as well as other consequences such as psychological and physiological consequences; for example, a recent study found a higher risk for individuals with problem gambling when it comes to sick leave (Berman et al., 2023b).

These negative consequences are also present for CSOs. A CSO experiences many of the harms previously described for a person who has gambling problems (Dowling et al., 2022; Irie & Kengo, 2022; Tulloch et al., 2023). CSOs experience a range of negative impacts, including significant financial issues, relationship difficulties, poorer emotional well-being as a result of worrying about the gambler and loss of trust. This is also true for children growing up with a parent who gambled (Dowling et al., 2022; Irie & Kengo, 2022; Tulloch et al., 2023). A study provides further evidence for the harm experienced by CSOs. They had significant financial issues, relationship difficulties, poorer emotional well-being because of worrying about the gambler and loss of trust (McCarthy et al., 2023). Adding to this, a recent Australian study found that the harms were strongly associated with high levels of distress and negative emotions. This, in turn, had an impact on the ability to function properly at work or to keep up other responsibilities (Tulloch et al., 2023). A significant difference, however, is that it is difficult for a CSO to perceive whether a person close to them gambles and, based on that, has problems. Therefore, finding out that an individual close to you has a gambling problem, and that there might be financial problems as a consequence of this, can be associated with stress and trauma. Regardless of whether the person is a partner, parent or child who has a problem with gambling, the relationship with the CSO deteriorates (Irie & Kengo, 2022; Tulloch et al., 2023).

Furthermore, Castrén et al. (2021) found that different subgroups of CSOs had different risk levels when it comes to experiencing different types of harm indicating that there is a need to distinguish between different subgroups of CSOs. Similar results were reported by Tulloch et al. (2023b), who found that harm differed between different types of CSOs where partners and ex-partners experienced the highest level of psychological distress and negative affect. This was followed by family members and non-members. Another study found that female CSOs were more likely to report harm from a partner or family member and male CSOs were more prone to report harm from a non-family member (Hing et al., 2022). Similar findings were found by Newall et al. (2024). Thus, it appears that levels of harm and negative consequences differ between different types of CSOs, as well as what type of relationship they have with the individual who gambles.

Another risk is that the relationship ends because of the CSO. In studies concerning relatives, it appears that problems with trust is a negative consequence that affects the relationship (Irie & Kengo, 2022). Being exposed to violence is also common not only for the person who has a gambling problem directed at CSOs, but also vice versa with CSOs who are violent towards the person who has gambling problems. Children also have an increased risk of being exposed to violence (Irie & Kengo, 2022). Another consequence of having someone who has gambling problems in your vicinity is that the risk of one's children developing gambling problems is high (Irie & Kengo, 2022). The physical problems that affect CSOs comprise headaches (both temporary and chronic), stomach problems that include pain and back pain. The psychological problems that relatives experience are feelings of anxiety, depression, anger and isolation (Irie and Kengo, 2022). A Swedish longitudinal study investigated problems faced by CSOs. The problems that relatives face did not differ between men and women. The problem areas were financial problems, risky alcohol consumption, psychological problems and conflicts with other relatives. At the 1-year follow-up of the participants in the study, relatives still had a higher degree of psychological problems and at-risk drinking (Svensson et al., 2013).

Another important aspect regarding the health of CSOs is suicide. Several studies have investigated this with a focus on the individuals that gamble. However, Wong et al. (2014) examined both suicidal ideation for the individual who gambled as well as relatives and found that about 20% of people who gambled had suicidal ideation and about 0.6% of relatives reported suicidal ideation. If an individual who has a gambling problem also reported suicidal ideation, this will put further pressure on the relatives and affect their well-being. It is also important to examine the relative's suicidal thoughts to make adequate risk assessments. One study found that children to gamblers had an increased risk of suicide attempts indicating that CSOs are at risk (Black et al., 2015).

There are different interventions available for CSOs. Helplines are available in many countries, as well help in a social service setting and different types of web-based counselling. However, the availability of psychological interventions is low overall (Dowling et al., 2022). Overall, between 10% to 25% of CSOs seek some form of help (Rodda & Lubman, 2014; Rodda et al. 2013; Svensson et al., 2013; Wood & Griffiths, 2007). A study on web-based counselling found that easy access was a reason for choosing web-based counseling and that they wanted help for themselves or trying to seek support for their relatives that gambled (Rodda et al., 2013). This was also found in a report from the Finnish helpline (Jaakkola et al., 2012; Pajula & Escartín, 2013). Another study found that the reasons for help seeking were concerns the gambling might become a major problem, negative emotions, difficulties maintaining normal daily activities, concerns for others and health concerns. Barriers found for help seeking were a desire to solve the problem on their own and shame (Hing et al., 2013). Furthermore, many CSOs attempt to solve the problem themselves using a range of coping strategies (Dowling et al., 2022). Also, CSOs might at first attempt to help the gambler and not themselves, leading to an increase in problem severity for themselves (Rodda et al., 2013). It is thus important to understand what type of help different categories of CSOs are interested in.

A handful of studies have investigated support for CSOs (Edgren et al., 2022). In broad terms, these studies have had two aims: (1) to involve the CSO to help the individual with gambling related problems to reduce or quit gambling through supporting self-help strategies and/or to seek formal treatment or (2) to support CSOs in their own right; for example, by working with coping strategies, psychoeducation about gambling and PG or reducing depression and anxiety. This also reflects what is known about the preferences of CSOs from previous studies (Rodda et al., 2020). In a recent meta-analysis of interventions involving CSOs, seven studies were found eligible of which three were CSO directed, four were couple oriented and two were low threshold online interventions (Edgren et al., 2022). The results have been mixed, at best, when it comes to motivating the individual with PG to seek treatment, whereas supporting CSOs has been met with some success (Edgren et al., 2022). A few studies have also investigated the role of CSOs in treatment for PG, with predominantly positive results. The mode of delivery has ranged from rather intense face-to-face couple treatments to more low-threshold alternatives, such as online self-help. However, many CSOs refrain from seeking help because of embarrassment, guilt, shame and stigma (Hing et al., 2013), although research suggests that, when they do, they prefer low-intensity interventions such as telephone counseling, self-help and online support (Hing et al., 2013).

The need for support for CSOs decreased during the COVID-19 pandemic in Finland (Marionneau & Järvinen-Tassopoulos, 2022) indicating that the consequences for CSOs when gambling is limited comprise a lower level of harm. However, a study investigated the number of calls to helplines in Sweden, Denmark and Finland found that there was only a decrease in helpline calls in Finland (Wall et al., 2023). This difference alongside the previously referenced Finnish study might reflect that gambling in Finland was more restricted because individuals with a gambling problem in Finland more frequently use electronic gambling machines.

Helplines are frequently used intervention sought by CSOs (Dowling et al., 2022). Research has shown that CSOs constitute around 40% of those who contact helplines (Bastiani et al., 2015; Potenza et al., 2001).

However, there is need for more real-world research on what types of problems the CSOs are facing and research on which problems are present for different types of CSOs (mainly parents or partners). Previous studies have presented data from questionnaires or qualitative studies based on self-reports. There is a need for research that presents the needs of CSOs when they are actively seeking help because this might provide a more accurate description of their situation. Therefore, our research tries to address an existing research gap in the field. Furthermore, a previous review concluded that there has been a focus on intimate partners and not so much research has been carried on other types of CSOs (Dowling et al., 2022). Therefore, gender and age are important aspects to focus on. In addition, more research is needed regarding CSOs who seek help because they have a relative or friend who has a problem with gambling. Gaining more insight regarding support-seeking CSOs could help tailor interventions that might decrease the burden of harm faced by this group.

## Aims of the study

The study aimed to investigate the overall characteristics and urgent problems for CSOs who seek help and guidance from the national Swedish helpline because of relatives who gamble.

The following research questions will be addressed:

What characterizes (e.g., gender, age) CSOs who contact the helpline?
Which problem areas do the CSOs experience and need help with because of having contact with an individual with gambling problems?
What type of help does the CSO want depending on how long they have known about the problem?
Which are the differences and similarities between different types of CSOs, such as relatives or partners?

## Methods

### Study setting, procedure and participants

The Swedish gambling helpline, Stödlinjen, is run by the Centre for Psychiatry Research at Stockholm County Council and funded by the Swedish Ministry of Health and Social Affairs. Stödlinjen was established in 1999. It provides support via phone and chat to individuals that gambles and their CSOs. In 2023, 1210 gamblers and 1120 CSOs contacted the helpline for counselling, around 20,000 individuals tested their gambling habits at the online problem gambling screener and the helpline webpage had more than 700,000 unique visitors. Around 15 counsellors trained in Motivational interviewing work at the helpline. Apart from being trained in gambling related issues, the counsellors receive regular training in adjacent areas that regularly comes up in the conversations such as, mental health, suicidal ideation, intimate partner violence and financial counselling. The helpline is open on weekdays and the opening hours vary during the week, the helpline opens between 9.00 h and 11.00 h, and closes between 15.00 h and 19.00 h, depending on weekday (Stödlinjen, 2024).

After each call or chat to the helpline, details regarding the content of the contact are recorded by the counsellors in an online database with pre-defined alternatives. The online database includes 267 pre-defined items covering both gamblers and CSOs. The items cover, among others, background information, which types of gambling that cause harm, experienced harm, reasons for contacting the helpline, focus for the conversation and which support that was offered. For this study, we focused on the items covering conversation focus and which type of support that the CSOs were offered. Because not all items are filled out after the conversations (e.g. age of the contactor rarely comes up in the conversations), some items have higher response frequency than others.

### Statistical analysis

Data are presented as numbers and percentages. To compare the frequencies of categorical variables, chi-squared tests were used. The variable type of CSO originally contained nine categories, which were collapsed into three categories. Four categories of relatives: “other relative”,” child”,” parent” and” sibling”) were collapsed into one category, “relatives”. The categories” grandparents” and “ex partners” were omitted due to few observations (*n* = 23 and *n* = 107, respectively) and, because they differed in terms of type of relationship with the gambler, they did not suite any of the three collapsed categories. This resulted in the three final categories: “relatives”, “partners” and “friends”. Because the data set contains missing data in most variables, given the nature of the data collection, the actual number of observations for each variable is presented in the results section.

### Ethical considerations

The study was approved by the Swedish Ethical Review Authority (Dnr 2022-03651-01). All collected material was processed confidentially in coded form. Data were handled and was stored following the European General Data Protection Regulation (GDPR).

## Results

### Characteristics of the sample

The total sample consisted of 4111 participants. The gender distribution of the sample was 87.1% females ( Table 1.). There were a majority of females for the CSOs who were partners or relatives and for friends the ratio was almost 35% who were females. Most of the partners live with a person who has a gambling problem, but this is not the case for other relatives and, not surprisingly, friends. Most of the gamblers have known about the problem for less than a month or over a year.

**Table 1.** Participant's characteristics, reported in the helpline contacts (*n* = 4111).

	Partner (*n* = 1486) (36.1%)	Relative (*n* = 2243) (54.6%)	Friend (*n* = 382) (9.3%)	χ^2^	*p*-value
CEOs gender*	*n* = 1243	*n* = 2072	*n* = 312		
Woman	1083 (87.1%)	1435 (69.3%)	109 (34.9%)	315.1**	< .001
Man	158 (12.7%)	633 (30.6%)	202 (64.7%)		
Other gender alternative	2 (0.3%)	4 (0.2%)	–		
Gamblers gender	*n* = 1468	*n* = 2227	*n* = 373		
Man	1262 (85.9%)	1846 (82.9%)	300 (80.4%)	9.2**	0.010
Woman	192 (13.1%)	375 (16.8%)	58 (15.6%)		
Other gender alternative	14 (0.9%)	6 (0.3%)	15 (4.0%)		
CSOs co-habiting with gambler	*n* = 1273	*n* = 1765	*n* = 282		
Yes	1125 (88.4%)	308 (17.5%)	6 (2.1%)	1752.5***	< .001
No	115 (9.0%)	1388 (78.6%)	276 (97.9%)		
Partly	33 (2.6%)	33 (2.6%)	–		
Time-period CSOs known about the problem gambling	*n* = 951	*n* = 1421	*n* = 158		
Less than a month	388 (40.8%)	452 (31.8%)	64 (40.5%)	28.9	< .001
Less than a year	143 (15.0%)	193 (13.6%)	25 (15.8%)		
More than a year	420 (44.2%)	776 (54.6%)	69 (43.7%)		
CSOs age (years)	*n* = 192	*n* = 260	*n* = 36		
Under 18	1 (0.5%)	8 (3.1%)	2 (5.5%)		
18–24	43 (22.4%)	27 (10.4%)	6 (16.7%)		
25–34	72 (37.5%)	48 (18.5%)	11 (30.6%)		
35–44	28 (14.6%)	28 (10.8%)	5 (13.9%)		
45–54	24 (12.5%)	29 (11.1%)	4 (11.1%)		
55–64	18 (9.4%)	56 (21.5%)	4 (11.1%)		
65–74	4 (2.1%)	52 (20.0%)	4 (11.1%)		
Over 74	2 (1.0%)	12 (4.6%)	–		

*Characteristics were not reported by CSOs in all helpline calls. **Category *other* was excluded as a result of few observations. ***Category *partly* was excluded as a result of few observations.

#### CSO problem areas

When it comes to problem areas for the CSOs and what they discussed during the phone calls, the three groups viewed seeking help as the most important feature. For partners, the second most important category was taking care of oneself. For relatives and friends, the second most important category was better communication and limit setting, which was also an important category for partners and came in third (Table 2.).

**Table 2.** Themes discussed in CSOs helpline contacts.

	Partner (*n* = 1486)	Relative (*n* = 2243)	Friend (*n* = 382)	χ^2^	*p*-value
Seeking help	708 (48%)	1265 (56%)	170 (45%)	37.3	< .001
Taking care of oneself	568 (38%)	416 (19%)	38 (10%)	235.4	< .001
Limit setting	505 (34%)	724 (32%)	74 (19%)	30.7	< .001
Communicate better	494 (33%)	696 (31%)	109 (29%)	3.9	0.15
Protect one's own economy	327 (22%)	292 (13%)	33 (9%)	70.6	< .001
Separation	321 (22%)	40 (2%)	5 (1%)	462.8	< .001
Limiting the possibility to gamble	263 (18%)	443 (20%)	78 (20%)	2.9	0.23
Take control of the gambler's economy	219 (15%)	360 (16%)	29 (8%)	18.5	< .001
Getting an overview of the gambler's economy	202 (14%)	299 (13%)	25 (7%)	14.8	< .001
Other measures	103 (7%)	195 (9%)	53 (14%)	18.9	< .001
Encourage the gambler to engage in non-gambling activities	100 (7%)	188 (8%)	30 (8%)	3.4	0.18

### How the CSO found out about the gambling problem

In about 40% of cases, the individual who had gambling problems told the CSO. However, in more than half of the cases for the partner-CSOs, they found out about the gambling and the extent of the problems by experiencing a lack of money, finding invoices or unpaid bills or finding gambling receipts. Because almost 85% of the partners live with the individual who has a problem with gambling, this indicates how many CSOs have been unaware of the problem even though they have lived together with the individual with the problem. Many of the relatives also experienced a lack of money (Table 3).

**Table 3.** How CSOs found out about the problem gambling (*n* = 2523).

	Partner (*n* = 918)	Relative (*n* = 1400)	Friend (*n* = 205)	χ^2^	*p*-value
The gambler told the CSO	364 (39.7%)	530 (37.9%)	93 (45.4.%)	0.3	0.84
Lack of money	341 (37.1.0%)	543 (38.8%)	73 (35.6%)	4.2	0.13
Found letters of demand/invoices	127 (13.8%)	103 (7.4%)	7 (3.4%)	37.5	< .001
Found gambling receipts	63 (6.9%)	23 (1.6%)	1 (0.5%)	–	–
Someone else told the CSO	23 (2.5%)	201 (14.4%)	31 (15.1%)	87.4	< .001

### Differences and similarities between different types of CSOs when it comes to how long they have known about the gambling problems and type of support they need

Regardless of how long the CSO had known about the problematic gambling, trying to get help for the individual with the problem was the most prevalent cause for calling the helpline. Taking care of oneself was second most feature for partners regardless of how long they had known about the problem, but, for relatives, taking care of oneself became more important after they had known about it for over a year indicating diverse needs for relatives. Separation was more prevalent for partners than for relatives and friends, indicating that it is more stressful and harder for partners to keep on having a relationship with the individual who has the problem. Communicating better was an important aspect for all of the separate groups of CSOs (Table 4).

**Table 4.** What the CSOs wants help with* and how long they have known about the problem.

	Partner			Relative			Friend			χ^2^	*p*-value
Time the CSO has known for about the problem	<1 M *n* = 388	<1 Y *n* = 143	>1 Y *n* = 420	<1 M *n* = 452	<1 Y *n* = 193	>1 Y *n* = 776	<1 M *n* = 64	<1 Y *n* = 25	>1 Y *n* = 69		
Type of help requested											
Seeking help *n*, (%)	194 (50.0)	62 (43.4)	217 (51.7)	282 (62.4)	107 (55.4)	455 (58.6)	36 (56.2)	13 (52.0)	33 (47.8)	12.3	.015
Taking care of oneself	163 (42.0)	58 (40.6)	212 (50.5)	70 (15.5)	33 (17.1)	216 (27.8)	5 (7.8)	4 (16.0)	12 (17.4)	28.6	<.0001
Limit setting	126 (32.5)	68 (47.6)	193 (46.0)	132 (29.2)	72 (37.3)	346 (44.6)	12 (18.8)	13 (52.0)	24 (34.8)	20.6	<.0001
Communicate better	127 (32.7)	68 (47.6)	154 (36.7)	164 (36.4)	84 (43.5)	259 (33.4)	21 (32.8)	9 (36.0)	32 (46.4)	4.5	.34
Protect one's own economy	98 (25.3)	34 (23.8)	121 (28.8)	43 (9.5)	28 (14.5)	154 (19.8)	11 (17.2)	3 (12.0)	9 (13.0)	27.9	<.0001
Separation	71 (18.3)	34 (23.8)	138 (32.9)	5 (1.1)	3 (1.6)	22 (2.8)	1 (1.6)	1 (4.0)	1 (1.4)	–	–
Limiting the possibility to gamble	93 (24.0)	31 (21.7)	69 (16.4)	109 (24.1)	47 (24.4)	139 (17.9)	14 (21.9)	7 (28.0)	11 (15.9)	8.3	.08
Take control of the gambler's economy	72 (18.6)	29 (20.3)	72 (17.1)	96 (21.2)	44 (22.8)	124 (16.0)	9 (14.1)	3 (12.0)	6 (8.7)	2.5	.64
Getting an overview of the gambler's economy	73 (18.8)	31 (21.7)	65 (15.5)	85 (18.8)	43 (22.3)	100 (12.9)	9 (14.1)	2 (8.0)	5 (7.2)	3.3	.51
Encourage the gambler to engage in non-gambling activities	30 (7.7)	15 (10.5)	34 (8.1)	59 (13.1)	24 (12.4)	63 (8.1)	8 (12.5)	3 (12.0)	10 (14.5)	.48	.98

*The CSOs can discuss several things that they need help with during the phone call. M = month; Y = year.

## Discussion

The aim of the study was to explore the characteristics of CSOs calling a helpline because they had an individual close to them who has a gambling problem. The results of the demographic data showed that almost all of the callers (90%) were either partners or parents (relatives). Also, about the same percentage (90%) of the callers co-habited with the individual who experienced gambling problems. Furthermore, a large part of the CSOs learned about the problems from a lack of money and finding payment notices and by the individuals with the gambling problem telling them about it. The CSOs that called had also known about the problems for different amounts of time. The two major groups were individuals who had known for less than a month and more than a year. The need for partners and relatives differed in relation to how long they had known about the problem.

In our study, the majority of contacts were with relatives who were not partners. This differs from results from some previous studies, where partners were generally more common among CSOs contacting help-services (Hodgins et al., 2007; Rodda et al., 2013), but similar to results from other telephone counseling services for gambling where partners make up approximately 34% of CSOs (Berman et al., 2023a). The shift in our study from partners to relatives (where parents make up the largest groups) may mean that new ways of helping the caller are needed. A previous review found that the experience and interpretation of the experience of having a relative that gambles is not the same for everyone in the family (Kourgiantakis et al., 2013), which indicates a need for different strategies.

Another important finding was that half of the partners in the sample found out about the gambling problems via bank statements and finding gambling related material. Finding out about the problem in such a manner might produce a shock for, in most cases, a partner to the individual who has a problem. This was also the case in previous studies where the CSOs found out at a late stage about the problems (Orford et al., 2017; Sullivan et al., 2007). More effort might be needed to provide services for these individuals because delayed interventions might produce negative emotions such as anxiety and depression in a subsequent stage. Psychosocial interventions are always important in trying to help a family that is exposed to an individual who has gambling problems. However, providing help for significant other that found out in this manner might be an important step to prevent severe consequences including mental illness.

The themes that were discussed during the phone calls mirror results from previous studies, where CSOs tend to want both gambler-focused advice and support regarding their own situation (Rodda et al., 2020). The most commonly sought advice was on where to seek treatment or support for the person who experienced gambling problems, followed by several themes reflecting a focus on the well-being of the CSO. One important aspect is that individuals that gamble are not prone to seeking help (Bijker et al., 2022) and CSOs might feel the need to encourage the individual who gambles to seek help but do not know where to turn. As such, the helpline might function as a first step in trying to cope with a relative that gambles and help them to decrease their gambling. Similar findings were in evaluation of the Finish helpline (Jaakkola et al., 2012; Pajula & Escartín, 2013).

Furthermore, one important aspect regarding the focus on the gambler from the perspective of the CSOs is that it might prevent CSOs from getting help for their own experienced harms. If CSOs abstain from getting help with their problems, there might be a risk that their physical and mental health deteriorates. In light of this, suicidality and long-term illness might be a risk for CSOs. Again, understanding the needs of different types of CSOs might be helpful to understand which services are necessary to provide. Furthermore, another important aspect when supporting a CSO is to have information about counselling and treatment available for the individual who gambles and try to establish contact with that individual to take some of the pressure away from the CSO.

Many interventions and support options for gambling CSOs, such as Community and Reinforcement Therapy and Family Training (CRAFT) and Behavioral Couples Therapy (BCT) evolved from couple-focused therapies, and there is a risk that these methods do not fully cater to the needs of other types of CSOs. Thus, there is a need to adapt current strategies regarding CSOs to also incorporate strategies for relatives. To date, there are not many options for relatives to get help if they have a relative who has a gambling problem. Another important dimension is that almost all of the relatives do not live with the individual who is experiencing problems. This needs to be taken into account when planning interventions for relatives. The CSOs wanted to help the individual who gambles by getting them help and also looking to communicate better. Creating interventions that could ameliorate communication would be one option to help CSOs that are relatively cost-effective.

A usually important theme in interventions such as CRAFT and BCT (i.e. to encourage the gambler to engage in other activities) was the least endorsed item in this study. This could be because many CSOs contacting the helpline are in a state of crisis, and thus not particularly interested in interventions that would demand patience and rigorous planning. It could also reflect the limits of anonymous helplines where the typical caller is making a single contact, making it difficult to initiate more exhaustive tasks. This could also explain why relatively few discussed issues related to economic issues, despite financial distress being a very commonly experienced harm among CSOs (Langham et al., 2016; Riley et al., 2018). In a Swedish setting, CSOs have the possibility to contact social services to get financial aid, which might explain the absence of discussing financial issues during the call. There is also a possibility to get counselling as an addition to the other support given by the social services. However, that counselling is mostly directed towards the gambler and that service is not widely available. That can partly explain why the callers did not show much interest in other interventions. For partners, taking care of oneself was an important aspect, as well as indicating that strategies for self-help are an important aspect when planning interventions for partners.

### Practical implications

The implications of these results mainly concern the difference in what themes are discussed and how the CSOs found out about the gambling problems. The first result highlights the different groups of CSOs and thus the different needs of the CSOs during the phone call and this affects the interventions that might need to be offered. This is line with Castrén et al. (2021) who found different sets of subgroups in their study. Subgroups with different risks and needs associated with them calls for tailored interventions to address the various problems faced by CSOs. Adding to this, the way that the CSOs found out about the gambling problems is also a case for concern because of the potential shock and negative consequences. This is an important aspect when providing interventions and help for this multifaceted group.

Furthermore, a key aspect when talking to CSOs appears to be to help them find help for the individual who gambles and to improve communication. Time is also an important aspect because the needs appear to vary depending on how long the CSO has known about the gambling and associated problems. This is important because the review found that only one in 25 moderate risk gamblers and one out of five individuals with gambling problems seek help (Bijker et al., 2022). Finding help for CSOs on where to find help for the individual is also important in trying to lessen the burden of harm.

### Limitations

The main limitation regarding the present study has to do with the individuals calling the helpline. The CSOs that call the helpline might not be representative of the entire CSO-population. One plausible line of argument is that the CSOs calling the helpline are more prone to seeking help compared to the overall CSO-population or that parts of the rest of the population have social support or other ways to get support and therefore do not to call the helpline. This limits the conclusions that one can make on a general level for CSOs. However, given the large sample size, some of the demographics might be similar to the overall CSO-population.

Furthermore, social desirability might be a factor that can have influenced the data provided by the CSOs because CSOs might hesitate to disclose information about their state as a result of experiencing shame when discussing their problems with a person who they do not know. They could be prone to give an inaccurate view and a more positive view of their situation. The possibility of being anonymous might decrease this risk somewhat. They might also understate their problems and concerns during the call for the risk of being stigmatized.

Additionally, there are some gaps in the data that might have skewed the results. The data are gathered during and after the phone call by the personnel manning the helpline. In some cases, data were lost because the individuals managing the helpline forgot to ask or catalogue data from the CSOs. Thus, there is a lack of adherence when it comes to reporting data by the personnel managing the helpline. However, this limitation is mitigated by the large sample size and the fact the data missing are not systematic.

### Future research

Future studies should focus on more comprehensive investigations of CSOs including main problem areas, subgroups of CSOs and different subgroup characteristics. Longitudinal studies are needed to understand how the situation of a CSO changes over time. Future research should also focus on which interventions are suitable for the entire group of CSOs and different subgroups of CSOs to be able to tailor interventions. Also, another important aspect is to compare data on CSOs from help lines from different countries to understand the needs across populations and countries.

## Conclusions

CSOs calling the Swedish helpline comprise a diverse population with different types of needs, as mirrored in the themes in the phone calls, what type of relationship that they had with the individual who gambles and how they found out about the problems. More information is needed to be able to understand the characteristics of different subgroups of CSOs and their needs to create effective interventions.
